# Achieving community-based postpartum follow up in eastern Uganda: the field experience from the MamaMiso Study on antenatal distribution of misoprostol

**DOI:** 10.1186/s13104-017-2849-5

**Published:** 2017-10-26

**Authors:** James Ditai, Laura J. Frye, Jill Durocher, Meagan E. Byrne, Sam Ononge, Beverly Winikoff, Andrew D. Weeks

**Affiliations:** 1 Sanyu Africa Research Institute (SAfRI), Mbale Regional Referral Hospital, P.O Box 2190, Mbale, Uganda; 2 Sanyu Research Unit, Department of Women’s and Children’s Health, University of Liverpool, Liverpool Women’s’ Hospital, Crown Street, Liverpool, L8 7SS UK; 3 Gynuity Health Projects, 15 East 26th Street, Suite 801, New York, NY 10010 USA; 4 Department of Obstetrics and Gynaecology, Makerere University College of Health Science, P.O Box 7072, Kampala, Uganda

**Keywords:** Follow up, Birth, Misoprostol, Self-administration, Uganda, Postpartum hemorrhage, Community-based

## Abstract

**Background:**

Advance provision of misoprostol to women during antenatal care aims to achieve broader access to uterotonics for the prevention of postpartum hemorrhage. Studies of this community-based approach usually involve antenatal education as well as timely postpartum follow-up visits to confirm maternal and neonatal outcomes. The MamaMiso study in Mbale, Uganda sought to assess the feasibility of conducting follow-up visits in the postpartum period following advance provision of misoprostol for postpartum hemorrhage prevention. MamaMiso recruited women during antenatal care visits. Participants were asked to contact the research team within 48 h of giving birth so that postpartum follow-up visits could be carried out at their homes. Women’s baseline and delivery characteristics were collected and analyzed with respect to follow-up time (‘on time’ ≤ 7 days, ‘late’ > 7 days, and ‘lost to follow up’). Every woman who was followed up late due to a failure to report the delivery was asked for the underlying reasons for the delay. When attempts at following up participants were unsuccessful, a file note was generated explaining the details of the failure. We abstracted data and identified themes from these notes.

**Results:**

Of 748 recruited women, 700 (94%) were successfully followed up during the study period, 465 (62%) within the first week postpartum. The median time to follow up was 4 days and was similar for women who delivered at home or in facilities and for women who had attended or unattended births. Women recruited at the urban hospital site (as opposed to rural health clinics) were more likely to be lost to follow up or followed up late. Of the women followed up late, 202 provided a reason. File notes explaining failed attempts at follow up were generated for 164 participants. Several themes emerged from qualitative analysis of these notes including phone difficulties, inaccurate baseline information, misperceptions, postpartum travel, and the condition of the mother and neonate.

**Conclusions:**

Keeping women connected to the health system in the postpartum period is feasible, though reaching them within the first week of their delivery is challenging. Understanding characteristics of women who are harder to reach can help tailor follow-up efforts and elucidate possible biases in postpartum study data.

*Trial Registration Number* ISRCTN70408620 December 28, 2011

## Background

Misoprostol is a low-cost, effective [1–4], and safe [5] intervention for the prevention of postpartum hemorrhage (PPH); however its usefulness is dependent upon its availability at the place and time of delivery. An integrative review of global implementation efforts suggests that self-administration of the medicine by women delivering at home is a way to achieve broader uterotonic coverage to prevent PPH [6]. To date, there are several published experiences with antenatal distribution of misoprostol to women for self-administration including efforts in Afghanistan [7], Nepal [8], Ghana [9], Liberia [10], Sudan [11], Nigeria [12], and Uganda [13].

Over two-thirds of the poorest women in sub-Saharan Africa give birth at home, and over half of their births are unattended [14]. Postpartum follow-up visits at home can help keep women connected to the health system. This strategy has been used in research to track the self-administration of misoprostol in order to provide insight into best distribution strategies and resource utilization. Additionally, these visits can provide data on a range of delivery outcomes for women in the community; such data can be helpful in the evaluation of efforts to improve health outcomes for women after childbirth.

In Uganda, the MamaMiso study was a randomized, placebo-controlled trial of antenatal distribution of misoprostol to women for self-administration to prevent postpartum hemorrhage [13]. One of the aims of the study was to assess the feasibility of conducting follow-up visits for data collection on maternal and neonatal outcomes in the immediate postpartum period and to track the distributed medicines. This analysis presents quantitative and qualitative findings on which women were successfully followed up and the self-reported descriptions of challenges associated with keeping connected to the study team during the immediate postpartum period.

## Methods

From May to October 2012, a study of self-administered misoprostol for PPH prophylaxis (MamaMiso) was implemented in Eastern Uganda enrolling 748 women. The main clinical findings are reported elsewhere [13].

Trained clinical staff recruited pregnant women into MamaMiso during antenatal care visits at four participating health facilities (one regional hospital and three health centers). The regional hospital was a tertiary facility located in the center of town, serving a population of about 4–5 million people from 14 districts and staffed by OBGYN specialists. The health centers were rural facilities staffed primarily by clinical officers and midwives. Women were only recruited to the study if they lived within one of the pre-specified 200 villages near to the recruiting centers.

During antenatal care visits, women with estimated gestational ages 34 weeks or higher who did not plan to travel after their delivery were informed about the study aims and procedures (details reported elsewhere). Women who consented to be involved in the study were asked for contact information including up to two telephone numbers and detailed directions to their place of residence (including a sketched map when possible). Baseline demographic data and reproductive histories were collected as well as an assessment of hemoglobin (Hb) levels. Women were provided with a neck purse containing the study medication (either misoprostol or placebo), instructions for its proper use, and a phone number to call to report their delivery. Women were asked to report their deliveries immediately either directly to the study team or to their village health teams (VHT) who would subsequently notify the study team. VHTs are composed of community volunteers who provide health information and linkages to health services.

Women were informed that a postnatal follow-up visit would be conducted at home 3-5 days after delivery, a period selected both to capture the nadir of the hemoglobin reading and to minimize poor recall of labor and delivery. In the three health centers, a VHT Coordinator was present at enrollment and linked the participants to VHTs who assisted with delivery notification. To incentivize timely notification of deliveries, VHTs were informed that they would receive 10,000 Ugandan Shillings (~ 4 USD) for the timely report of a delivery. The incentive was split between the women and VHT if both were involved in the delivery notification and a smaller incentive was provided for deliveries reported after 5 days. Efforts were made to follow up women, even beyond the target time period of 3–5 days postpartum in order to confirm the well-being of the mother and newborn. Upon successful follow up, women were provided a small gift of soap.

While the study aimed for follow up between 3 and 5 days, for the purposes of this data analysis a cut-off time of 7 days postpartum was used to make it comparable to other studies and research protocols (UNICEF and WHO recommend neonatal home based care within the first week of life [15]). Women were considered “on-time” if follow up was achieved within 1 week of the delivery date, “late” if they were successfully followed up 8 days or more after the delivery, and “lost” if the study team was never able to conduct a full follow-up interview. Some women who were “lost” were contacted by phone, and limited information was gathered on them. Patient characteristics and clinical outcomes were then analyzed by these ‘time to follow up’ categories.

Nearly all delivery notifications were received by the Research Fellow, mostly by phone. The Research Fellow then coordinated follow up by assigning a study staff member (midwife or nurse) to locate the woman, typically by using a motorcycle taxi to drive to the participant’s home. Follow up consisted of an in-person interview using a standardized case report form and the assessment of hemoglobin levels, using a Hemocue^®^ handheld device (Hemocue, Angelholm, Sweden). Information regarding the details of the delivery, medications used, and health status of the mother and baby were collected. Unused medicines or empty foil packs were also retrieved during the visit.

When follow-up difficulties arose early on, a system of keeping extensive file notes was put into place. Each woman who did not report her delivery on time was asked why, and when attempts by staff to follow up participants were unsuccessful, the details were noted.

This analysis seeks to understand the challenges of community-based follow up in the context of a placebo-controlled randomized trial of antenatal distribution of misoprostol to women for self-administration to prevent postpartum hemorrhage. Bivariate analyses of background and follow-up characteristics were conducted by follow-up time and multivariate logistic regression was used to predict follow-up category. Data were abstracted from the file notes and reviewed for initial and emergent themes using a general inductive approach. Themes were identified through an iterative process, and data were independently coded by two researchers (LF and MB).

## Results

Of the 748 women recruited, 700 women (93.6%) were successfully followed up. The median time to follow up was 4 days, ranging from 2 to 115 days postpartum. Nearly two-thirds (62.2%) of recruited women were followed up within 1 week postpartum and almost one-third after that (31.4%) with 48 women (6.4%) lost to follow up.

Bivariate analyses of participant baseline characteristics indicated that site of enrollment, age, and nulliparity were associated with follow-up time (Table 1). Multinomial regression revealed that only site of recruitment was significantly associated with follow up when all baseline characteristics were entered into the model. Women recruited at the hospital were more likely to be followed up late (p = 0.002) and were more likely to be lost to follow up (p < 0.001) than participants enrolled at health centers. Eleven percent of women recruited from the hospital site were lost to follow up compared to 1–4% recruited from the three health centers. Furthermore, the median time to follow up for women enrolled at the hospital was 7 days versus 4 days for the other recruitment locations (p < 0.001).

**Table 1:** Baseline characteristics by follow up time N (%) or mean [SD]

Baseline participant characteristics n = 748			
	On time follow up^a^ (n = 465)	Late follow up (n = 235)	Lost to follow up (n = 48)
Place of enrollment^b^			
Hospital	138 (29.7)	137 (58.3)	35 (72.9)
Health centers	327 (70.3)	98 (41.7)	13 (27.0)
Mean age* (years)	27.0 [6.7]	25.4 [5.5]	24.08 [5.6]
Nulliparous^b^	86 (18.5)	60 (25.5)	18 (37.5)
Primary occupation			
Housewife	349 (75.1)	178 (75.7)	34 (70.8)
Employed	99 (21.3)	54 (23.0)	12 (25.0)
Unemployed/student	17 (3.7)	3 (1.3)	2 (4.2)
Level of education completed			
No education	33 (7.1)	15 (6.4)	3 (6.3)
Primary	266 (57.2)	111 (47.2)	22 (45.8)
Secondary or higher	166 (35.7)	108 (46.2)	23 (47.9)
Mean hemoglobin at recruitment (g/dL)	11.2 [1.4]	11.3 [1.4]	11.4 [1.5]

In multinomial logit regression of all baseline characteristics, only place of enrollment retained significance at p < 0.05 level in the model

* Represents statistically significant difference at p < 0.05 level among the groups for log transformed age using one-way ANOVA

^a^Defined as within one week of birth

^b^Represents statistically significant difference among the groups using Chi square at 0.05 level

Details related to the participants’ delivery were recorded regardless of delivery location and timing of follow up (Table 2). The proportion of women delivering at home was similar among those followed up on time versus late. Similarly, use of the study medication at the time of birth did not differ between these two groups. However, the proportion of women with postpartum anemia (Hb < 9 g/dL) was significantly higher among those followed up on time as compared to those followed up late (p < 0.001). Additionally, a larger portion of women followed up late reported a delivery complication when compared to those followed up on time, including neonatal death (4.3% vs. 2.6%, p = 0.254), consulting a healthcare provider in the postpartum period (3.8% vs. 2.6%, p = 0.357), being admitted to a health facility (2.6% vs. 1.5%, p = 0.378), having a surgery (1.3% vs. 0.2%, p = 0.113), having a blood transfusion (1.3% vs 0.2%, p = 0.113), or experiencing a postpartum infection of any type (6.8% vs 4.9%, p = 0.219) though these outcomes were rare and none of the differences were statistically significant.

**Table 2:** Delivery characteristics by follow up time N (%)

Delivery participant characteristics n = 700		
	On time follow up (n = 465)	Late follow up (n = 235)
Home delivery	195 (41.9)	104 (44.3)
Used study medication	256 (55.1)	141 (60.0)
Skilled birth attendant^a^	269 (57.8)	134 (57.0)
Postnatal anemia (Hb < 9 g/dL)^b^	52 (11.2)	9 (3.8)
Self-reported heavy bleeding by pictorial	33 (7.1)	14 (6.0)
Neonatal death	12 (2.6)	10 (4.3)
Maternal complication^c^	30 (6.5)	18 (7.7)

^a^Defined as a doctor, midwife, or trained nurse

^b^Statistically significant difference using Chi square test at 0.05 level

^c^Maternal complications include: postpartum infection, postpartum consultation with health professional, postpartum admission to a health facility, surgery, and transfusion

### Themes explaining late follow up

Of the 235 women who were followed up outside of the 7-day timeframe, 195 (82.6%) either notified the study team of their delivery late, or not at all. In fact, for 21 participants, follow up took place as a result of the proactive calculation of expected delivery date by the study team and subsequent phone calls or home visits to participants. Of participants followed up late, 202 supplied a reason (or several) for their lack of timely notification in response to an open-ended question.

While the quantitative data elucidates which women were more difficult to follow up, the qualitative data reveals several themes about why some of these difficulties arose. Five main themes emerged: phone difficulties, inaccurate baseline information, misperceptions, travel in the postpartum period, and the condition of the mother and neonate. Table 3 contains illustrative quotations related to each theme.

**Table 3:** Illustrative quotations

THEME: phone difficulties	“My phone was faulty, the battery was spoiled so I was off air” *(Quotation from woman)*
	“I delivered at home and did not report because I did not have the phone because my husband had gone for a safari and had not come back…” *(Quotation from woman)*
	“My husband does not allow me to make phone calls.” *(Quotation from woman)*
	“I left the book which had your telephone number in the hospital. I did not remember about the telephone number on the participant information sheet.” *(Quotation from woman)*
THEME: inaccurate baseline information	“I followed up the participant at the given address but failed to get her because the villages given don’t exist.” *(Quotation from study staff)*
	“The [name of contact person provided] had died some time back, [the neighbors] told me that they had never come across the name of the participant in their village.” *(Quotation from study staff)*
	“I couldn’t locate [the participant] and none of the residents from the shops could either…the residents directed me to the local chairperson’s home who also didn’t know this participant.” *(Quotation from study staff)*
THEME: misperceptions	“I thought [reporting my delivery] was not important since I was fine after delivery.” *(Quotation from woman)*
	“I delivered and forgot to call until a nurse called me and asked if I had delivered.” *(Quotation from woman)*
	“Participant followed up late because she feared calling us due to the fact that she swallowed only two tablets and missed one… She thought we would blame her for not swallowing the third tablet.” *(Quotation from study staff)*
	“[The husband] answered the phone … He wondered whether it was necessary to follow [the wife] up… He said he will need us to follow her up in his presence… He also asked me why a man called to find out whether she had delivered.” *(Quotation from study staff)*
THEME: travel/events	“On my third day after delivery I lost someone and travelled… for a burial. That’s why the MamaMiso staff were not able to find me.” *(Quotation from woman)*
	“When my baby died, I lost my marriage, that’s why you were not able to get me at my home. I had come here to stay with my mother.” *(Quotation from woman)*
	“When I reached the home, I did not get the participant because she had lost her one twin and they had taken the twin to another village for burial.” *(Quotation from study staff)*
	“I was told by the neighbor that she left for their village… after delivery. She went with her mother because they had a misunderstanding with her husband.” *(Quotation from study staff)*
THEME: condition of mother/neonate	“I was very sick and confused so I could not remember to report to you people that I had delivered.” *(Quotation from woman)*
	“After the death of the baby I was disturbed in the mind so I could not even remember to notify you that I had delivered.” *(Quotation from woman)*
	“I called the participant today… she said she was operated on and the baby passed on…she went to a relatives place for more support.” *(Quotation from study staff)*

Participant numbers and locations have been redacted from quotations. Quotations from women were typically provided in one of the three local languages (Ateso, Lugisu, Lugwere) and translated by study staff into English for recording. The English grammar of some quotations has been amended to facilitate comprehension

#### Phone difficulties

Most women (70.3%; n = 526) provided at least one phone number during recruitment, and 29.3% (n = 219) provided two phone numbers. However, among women who reported a reason for not notifying the study team of their delivery, the most common reasons were phone related (n = 68) including a lack of airtime, lack of reception, and batteries dying. Additionally, some women (n = 31) reported losing the study phone number to call to report their deliveries and others (n = 18) reported that they attempted to reach the study team by phone but were unsuccessful.

Women explained that it was not enough to have access to a phone as there were also important conditions for its use. The absence of a husband was reported by 10 women as a factor contributing to failed delivery notification, either because he was the only person with a phone or because phone use in his absence was not permitted.

#### Inaccurate baseline information

At enrollment, estimated gestational age (GA) (based on last normal menstrual period and clinical assessment) was recorded and the expected date of delivery calculated for each study participant. Eighty-seven women passed their expected date of delivery by more than 2 weeks, suggesting inaccuracies in the estimated GA at enrollment. This resulted in 22 additional home visits to women who were still pregnant, requiring an unplanned use of resources (motorcycle fare and staff time).

Poorly described or possibly false addresses were also evident in the study staff follow-up notes. The study contact/address form asked for extensive information on how to reach the woman’s home. However, upon attempting follow up, staff reported 35 instances when this information appeared to be fabricated, and the women were not known within the village that they had reported as their place of residence.

#### Misperceptions

Of the 202 women who failed to notify the study staff of their deliveries and provided a reason why, 49 stated that they “forgot” and an additional 41 women thought it was unnecessary to inform the study team of the birth. There were two women who attributed the failure to report their deliveries to “fear” because they had not fully complied with the study instructions. Additionally, one participant reported that her neighbors discouraged her from contacting the study team due to suspicions of the research. The file notes from staff show that once again the role of husbands was important, because in seven cases husbands either refused follow up in their absence (which delayed the process) or were uncooperative or openly hostile to the study team.

#### Travel/events

Of the 48 women lost to follow up, 26 traveled outside of the study area postnatally as reported by the woman herself or her family and neighbors. Additionally, among the 195 women providing reasons for late delivery notification, traveling or moving were mentioned by 40. Reasons for postpartum travel included relocating to a family members’ home, traveling due to the death of a loved one, or marital discord.

#### Condition of mother/neonate

Self-reported maternal and neonatal complications including admission to a hospital, surgery, blood transfusion, infection, or retained placenta were rare among participants (outcomes unknown for those lost to follow up). However, the qualitative reports of 14 women suggest that experiencing poor health in the postpartum period impacted delivery notification and thus timely follow up.

## Discussion

In the MamaMiso study, follow up in the immediate postpartum period was feasible and a high follow-up rate (93.6%) was achieved. A similarly high follow-up rate [8] was achieved in the community misoprostol study in Nepal, though experiences in Nigeria and South Sudan reported rates much lower (84 and 76% respectively) [11, 12]. In this study, similar rates of follow up were achieved irrespective of place of the birth and the presence of a skilled birth attendant. This finding may suggest that even women who are disconnected from the health system at the time of delivery by giving birth at home and without a skilled attendant are still amenable to postpartum follow up.

MamaMiso found that the main impediment to timely postpartum follow up was late delivery notification. A study of neonatal postpartum visits in Bangladesh reported the same issue [16]. This experience showed that it was particularly effective to involve both women and VHTs in delivery notification and, as necessary, to seek women proactively by calculating expected delivery dates and by scanning health center registries for births.

Enrollment location appeared closely related to the success of follow-up visits. The study team hypothesized that participants recruited from the hospital were more difficult to follow up for a variety of reasons. First, hospital enrollees were urban women with fewer community connections and no VHT and thus study staff could not benefit from the assistance of a network of close-knit neighbors when searching for a participant. Another hypothesis is that women enrolling at the hospital were from farther reaches of the district and more likely to give false addresses both to qualify for antenatal care at the hospital and to qualify for the study. Last, the study team felt that urban addresses were inherently more difficult to locate due to the density of the housing and the transience of the populations. This finding has implications in light of the increasing urbanization of world populations and while some studies assume urban women have better access to care, this experience implies that they may in fact be more isolated or difficult to reach.

**Fig. 1:**
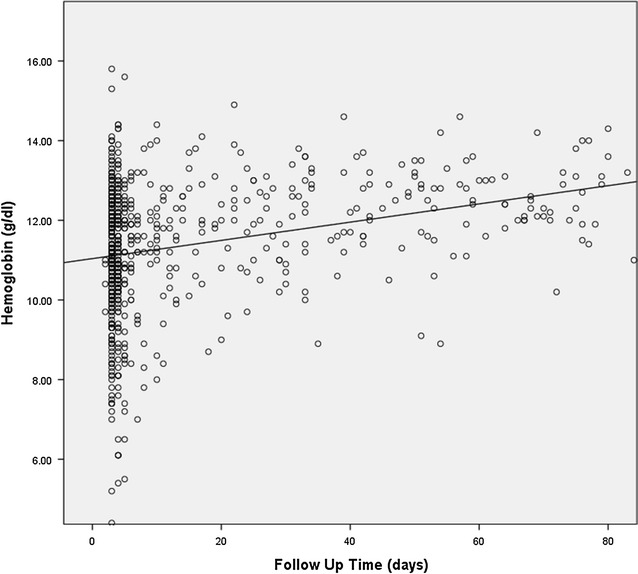
Postpartum hemoglobin values for all women followed up irrespective of place of delivery and use of study medication. This figure depicts a scatter plot with the follow up time in days on the x-axis and the hemoglobin value in g/dL along the y-axis and incorporates a linear best fit line. Line represents a linear best fit line for the full data set

Late follow up may have a number of effects on outcome data integrity. The recall of delivery outcomes might be related to the timing of follow up, causing a different degree of accuracy between the ‘on time’ and ‘late’ follow-up groups; self-reports of complications following delivery reflect a longer period of time when women were followed up late; and hemoglobin assessments for those followed up late are subject to the natural rebound in the postpartum period (Fig. 1). Our data do show higher rates of anemia detected among women followed up within 7 days, despite no differences in use of study medicine, antenatal hemoglobin levels, or self-reported excessive bleeding. It is plausible that women who experienced the symptoms of postpartum anemia were compelled to contact the study team at a higher rate than those without such symptoms or it is similarly plausible that late follow up missed measuring the nadir of hemoglobin levels, thus misclassifying women who would otherwise have been considered anemic. However early follow up may also have over identified anemia. Hemoglobin levels naturally rise postnatally due to the loss of the excess body fluid of pregnancy (diuresis). Future studies should consider follow-up timing differences as areas for potential bias when interpreting postpartum findings.

The qualitative data shows that the postpartum period can be a tumultuous one characterized by health complications, life events, and travel; therefore some loss to follow up must be anticipated. Some of the barriers encountered, like family deaths requiring travel, were inevitable but others can be addressed through improvements in research design for example recruiting women from their homes instead of antenatal clinics. Additionally, despite informed consent and enrollment, some women may not wish to be followed up. Their rights as participants must be respected.

A strength of this work is that few studies have such extensive qualitative details on the reasons for successes and failures of postnatal follow up. This analysis is limited by the non-systematic nature of the qualitative data and is therefore best used for hypothesis generating and to extract lessons learned. Furthermore, as these data were self-reported, interpretations of the data may be limited by the truthfulness of explanations provided.

## Conclusions

The strategies employed and lessons learned from the experience of MamaMiso can be applicable to a wide range of studies seeking to collect data on maternal or neonatal outcomes at the community level.

Keeping women connected to the health system in the postpartum period is feasible. The MamaMiso study showed that women who delivered at home and without a skilled birth attendant were equally likely to be followed up on time as those with institutional and attended deliveries. Women who enrolled during antenatal care at an urban hospital were especially difficult to follow up on time. In their own words, women explain that phone difficulties, misperceptions, postpartum travel, and condition of the mother or neonate also interfere with postpartum visits. Some of these issues can be ameliorated, though others are inevitable. Consideration of which groups of women are harder to reach in a timely matter is important when interpreting study findings based on postpartum assessments.

## Abbreviations

ANC: antenatal care
GA: gestational age
Hb: hemoglobin
OBGYN: Obstetrician–Gynecologist
PPH: postpartum hemorrhage
VHT: village health team
